# Expression pattern and regulation of genes differ between fibroblasts of adhesion and normal human peritoneum

**DOI:** 10.1186/1477-7827-3-1

**Published:** 2005-01-10

**Authors:** Ujjwal K Rout, Ghassan M Saed, Michael P Diamond

**Affiliations:** 1 Division of Reproduction Endocrinology and Infertility, Department of Obstetrics and Gynecology, Wayne State University, School of Medicine, Detroit, MI 48201, USA; 2 Division of Pediatric Surgery, Department of Surgery, University of Mississippi Medical Center, Jackson, MS 39216, USA

## Abstract

**Background:**

Injury to the peritoneum during surgery is followed by a healing process that frequently results in the attachment of adjacent organs by a fibrous mass, referred commonly as adhesions. Because injuries to the peritoneum during surgery are inevitable, it is imperative that we understand the mechanisms of adhesion formation to prevent its occurrence. This requires thorough understanding of the molecular sequence that results in the attachment of injured peritoneum and the development of fibrous tissue. Recent data show that fibroblasts from the injured peritoneum may play a critical role in the formation of adhesion tissues. Therefore, identifying changes in gene expression pattern in the peritoneal fibroblasts during the process may provide clues to the mechanisms by which adhesion develop.

**Methods:**

In this study, we compared expression patterns of larger number of genes in the fibroblasts isolated from adhesion and normal human peritoneum using gene filters. Contributions of TGF-beta1 and hypoxia in the altered expression of specific genes were also examined using a semiquantitative RT-PCR technique.

**Results:**

Results show that several genes are differentially expressed between fibroblasts of normal and adhesion peritoneum and that the peritoneal fibroblast may acquire a different phenotype during adhesion formation. Genes that are differentially expressed between normal and adhesion fibroblasts encode molecules involved in cell adhesion, proliferation, differentiation, migration and factors regulating cytokines, transcription, translation and protein/vesicle trafficking.

**Conclusions:**

Our data substantiate that adhesion formation is a multigenic phenomenon and not all changes in gene expression pattern between normal and adhesion fibroblasts are the function of TGF-beta1 and hypoxia that are known to influence adhesion formation. Analysis of the gene expression data in the perspective of known functions of genes connote to additional targets that may be manipulated to inhibit adhesion development.

## Background

Peritoneal adhesions resulting from surgical injury are often associated with pelvic pain, bowel obstruction and infertility [1]. Epidemiological studies conclude that 30 to 35% of all hospital readmissions are associated with adhesion associated complications, of which 4.5 to 5.1% are directly related to adhesions [2]. Mechanisms of adhesion formations are not completely known. It is also not clear why adhesion form in some patients and not in others. Therefore, deciphering genetic components that signal adhesion formation may help diagnose adhesion-prone patients prior to surgery. Needless to mention that such information will facilitate finding ways to prevent post-surgical adhesion formation.

Parietal and visceral peritoneum that surfaces the intraperitoneal organs is covered by a layer of squamous epithelial cells, the mesothelium. The submesothelial layer consists of fibroblasts, macrophages and blood vessels. Surgical abrasion to the peritoneum releases mesothelial cells, macrophages, fibroblasts, and blood containing cytokines and several cell types at the site of injury. Coagulation of blood creates a fibrinous mass between injured surfaces. In some patients fibrinolysis of clot followed by proliferation of mesothelial cells covers the wound. In others, failure of fibrinolysis followed by proliferation and migration of fibroblasts into the proteinous mass generates fibrous tissues of adhesion. Consequently, the process of adhesion formation include inflammatory response, fibrin deposition, cell-proliferation, -differentiation, -migration, -death, angiogenesis, extra cellular matrix (ECM) turnover regulated by cytokines, hypoxia, genetic and epigenetic factors [3].

Recent studies illustrate roles of peritoneal fibroblasts in adhesion development [4-10]. It is also proposed that fibroblasts from the chronic wounds migrate into the fibrin deposit; secrete ECM proteins causing wound contraction and scar formation [11]. The migration of fibroblasts may be coordinated by TGF-β1 mediated interactions of integrin receptors [10] with the RGD sequence of the fibrin, fibrinogen and fibronectin at the fibrin clot [12]. Additional cytokines and the hypoxic condition at the site of injury may also influence peritoneal fibroblasts to attain a phenotype supporting formation of adhesion tissue. This change in the phenotype of fibroblasts may be induced by changes in expression pattern of several genes during the process of adhesion development. Therefore, identifying differences in the global gene expression pattern between normal and adhesion fibroblasts may provide additional clues to the mechanisms by which normal fibroblasts attain the adhesive, proliferating and migratory phenotype required for the formation of fibrous tissues of adhesions. In the present study, we compared gene expression patterns between adhesion and normal peritoneal fibroblasts using GF211 gene filters (Research genetics) containing 4325 randomly selected known genes. Furthermore, we confirmed the expression pattern of genes of interest by a semiquantitative RT-PCR method and examined possible contribution/s of TGF-β1 and hypoxia in the transformation of normal peritoneal fibroblasts into an adhesion phenotype.

## Methods

### Peritoneal-tissue collection, fibroblast-isolation and culture

Tissues were collected at the initiation of surgery and after the entry into the abdominal cavity of female patients (25–50 years) undergoing laparatomy for pelvic pain as described earlier [4]. All patients gave informed written consent for the tissue collection, which was conducted under a protocol approved by the Wayne State University Institutional Review Board. Normal parietal peritoneal tissues were collected from these patients from the anterior abdominal wall, approximately midway between the umbilicus and symphyses pubis, and lateral to the midline incision. Peritoneal tissues from adhesions, that were at least 3 inches away from the site of normal tissue collections, were also collected from the same patient. The peritoneal fibroblasts were isolated and separated from mesothelial cells by a differential centrifugation procedure that is briefly described earlier [4]. The isolation of fibroblasts from mesothelial cells were also verified by the RT-PCR detection of Collagen type I, Matrix metalloproteinase-2 (MMP-2) and Transforming growth factor-β3 (TGF-β3) [13-15].

The primary cultures were maintained in a humidified incubator (37°C, 5% CO_2_) for 3 days in DMEM (Life Tech.) supplemented with 10% fetal bovine serum (Life Tech.) and antibiotics (Penicillin and Streptomycin 50 U/ml; Life Tech.). The monolayer of cells were passaged in trypsin-EDTA solution (Life Tech.). Cells at 3–5 passages were cultured in serum free medium in 75 cm^2 ^flasks (Fisher Scientific, Pittsburgh, PA) to 75% confluency prior to studies.

### Gene expression pattern in the fibroblasts from adhesion and normal peritoneum

Total RNA was isolated from monolayer of fibroblasts at 12 h of culture in serum free medium using Trizol reagent (Invitrogen Inc.). Human Gene Filters (GF211; Research Genetics, Inc., Huntsville, AL) containing 4325 known human cDNA spots were used for the identification of differentially expressed genes between adhesion and normal fibroblasts from human peritoneum. Method suggested by the manufacturer was strictly followed. In brief, 10 μg of total RNA from monolayer cultures of fibroblasts were subjected to cDNA synthesis in presence of 10 μl ^33^dCTP (10 mCi/ml; ICN Radiochemicals, Irvin, CA). Radiolabeled cDNA was separated from the free nucleotides using Bio-Spin 6 chromatography column (Bio-Rad Laboratories). Gene filters were labeled as adhesion or normal fibroblasts and washed with 0.5% sodium dodecyl sulfate (SDS) prior to prehybridization. Individual membrane was transferred to separate roller tubes of the hybridization oven (Fisher Scientific, Inc., Pittsburg, PA), each containing MicroHyb hybridization solution (Research Genetics) supplemented with Human Cot-1 DNA (Life Technology) and Poly dA (Research Genetics). The membranes were rotated at 10 RPM and at 42°C for 2 h. Radio-labeled cDNA prepared from adhesion and normal fibroblasts total RNA was denatured by heating in a boiling water bath for 3 min. The denatured probes were then injected into the prehybridization solution containing respective membrane. The membranes were hybridized with respective probe for 18 h at 42°C. The hybridization solution was then replaced with washing solution (2 × SSC containing 1% SDS). The temperature of the oven was raised to 50°C and RPM of rotors was increased to 15. Membranes were washed for 20 minutes when washing solution was replaced with a batch of fresh and prewarmed (50°C) washing solution. Washing was continued for additional 20 min. A third wash was performed with 0.5 × SSC solution containing 1% SDS at 55°C for 15 minutes. Membranes with cDNA spots facing up were covered with Saran wrap and exposed to phosphor screen (Kodak) for overnight. The screen was scanned with a Phosphor Imager (Storm System; Amersham Biosciences Corp., Piscataway, NJ). After acquisition of signal intensities from the normal and adhesion fibroblasts of one patient, filters were stripped according to protocol and subjected to gene filter experiments with the RNA samples from a second patient and images were scanned. Tiff images obtained from normal and adhesion fibroblasts of two patients were analyzed using Pathway 4 software (Research Genetics) for the identification of differentially expressed genes between the normal and adhesion fibroblasts of each patient.

### Relative abundance of selected genes in the fibroblasts from adhesion and normal peritoneum

Steady-state levels of mRNA of selected genes that are known to have a role in cellular adhesion, proliferation, migration, apoptosis and demonstrating different expression levels between adhesion and normal fibroblasts in the gene filter experiments were verified further by a previously described semiquantitative RT-PCR method [16]. Total RNA (1 μg) from the monolayer culture of adhesion or normal fibroblasts was subjected to reverse transcription as described earlier. Complementary DNA (100 ng) was subjected to PCR amplification of the cDNA of interests in a 25 μl reaction mixture containing 50 mM Tris-HCl (pH 8.4), 50 mM KCl, 2.5 mM MgCl_2_, 0.2 mM dNTP, 0.5 U Taq Polymerase (all from Life Technology, BRL) and 1 μM each of sense and antisense primers. Primer sequences were determined using Primer3 software from the Internet . The control primers (*sense *5'-ggaggttcgaagacgatcag-3' and *antisense *5'-cgctgagccagtcagtgtag-3') were expected to provide an amplicon of 509 bp from human 18S ribosomal subunit cDNA (gi: 337376). Accession numbers of genes of interests are provided in Table 2 and nucleotide sequences of primers and expected size amplicons are provided in the Table 3. Each PCR cycle consisted of a hot start at 95°C for 1 min, followed by melting at 95°C for 30 sec, annealing at 58°C for 1 min and extension at 72°C for 1 min. At the end an extension reaction at 72°C for 10 min was performed.

**Table 2:** Genes differentially expressed in the adhesion fibroblasts and known to have roles in cell-adhesion, -proliferation, -migration, -differentiation and -death.

**Accession Number**	**Definition**	**Fold Change**		**Functions**
		**P1**	P2*	
***Increased in Intensity***				

gi:17986276	Collagen, type IV alpha 2	2.4	2.7	See Discussion
gi:4506760	S100 calcium-binding protein A10	2.3	2.7	See Discussion
gi:6679055	Nidogen 2	6.4	7.1	See Discussion
gi:14250074	Transmembrane 4 superfamily member 1	3.7	2.6	See Discussion
gi:4758081	Chondroitin sulfate proteoglycan 2	3.4	3.2	See Discussion
gi:187538	Metallothionein 1E	4.3	4.0	See Discussion
gi:4336324	Small membrane protein 1	2.4	2.6	Cell viability [54]
gi:17738299	Cyclin-dependent kinase inhibitor 2A	2.0	2.0	Cell proliferation [55]
gi:16359382	Nuclear receptor subfamily 4, group A	1.6	2.1	Antagonizes TNF-α induced apoptosis [56]
gi:40353726	Synaptopodin	2.9	2.4	Actin cytoskeleton dynamics [57]
gi:23398519	Vasodilator-stimulated phosphoprotein	1.5	2.1	Enhances actin based cell motility, Cytoskeltal dynamics [58]
gi:28329	α-Smooth muscle actin	3.0	3.2	Myofibroblast transformation [44]
gi:14574570	Bcl-2 related gene bfl-1	1.6	1.4	Anti apoptotic; inhibitor of p53 induced apoptosis
gi:796812	p53 tumor suppressor	1.5	1.6	Cell cycle arrest and apoptosis [52]

***Decreased in Intensity***				

gi:184522	Insulin-like growth factor binding protein 3	3.2	2.3	See Discussion
gi:4504618	Insulin-like growth factor binding protein 7	2.3	2.0	Growth suppressing factor [59]
gi:28610153	Interleukin 8	3.2	2.6	Inhibits fibroblast migration, delays wound healing, reduces wound contraction [60]
gi:4504982	Lectin, galactoside-binding, soluble 3 [galectin)	3.0	3.0	Tumor-suppressive and pro apoptotic [61]
gi:12803916	Gap junction protein, beta 1, [Connexin 32)	1.8	2.2	Tumor suppressive and Proapoptotic [62]
gi:14589894	Cadherin 5, type 2, VE-cadherin [vascular]	2.3	1.7	Down regulation associates with tumor metastasis, Initiates endothelial-mesenchymal transdifferentiation [63]
gi: 16198356	Lactotransferrin	2.2	2.1	Inhibits growth of malignant tumors. Elevated by high level of estrogen [64]
gi:21619838	Lipocalin 2, Oncogene 24p3	3.3	2.5	Proapoptotic [65]
gi: 23273645	Calponin 1, basic, Smooth muscle cell	1.7	2.5	Inhibits smooth muscle cell contraction and Tumor Suppressive [66]
gi:40225461	RAP1A, member of RAS oncogene family	1.8	1.6	Inhibits cell proliferation [67]
gi:4507112	Synuclein-gamma	1.5	1.3	Expression reduced in carcinoma [68]

* Adhesion/Normal peritoneal fibroblasts values of gene expression intensity from patient 1 (P1) and 2 (P2).

**Table 3:** PCR primers, amplicon size and expression ratios of genes between adhesion and normal peritoneal fibroblasts

Transcripts	Primer Sequence (5' to 3')		Amplicon size (base pairs)	Adhesion/Normal Ratio Gene Filter*	Adhesion/Normal Ratio (RT-PCR)**
18S Ribosomal Subunit	Sense	ggaggttcgaagacgatcag	509	(No spot)	0.9
	Antisense	cgctgagccagtcagtgtag			
Collagen type IV alpha 2 chain(COL4A2)	Sense	caccatgcccttcctgtact	351	2.6	2.3
	Antisense	ttgcattcgatgaatggtgt			
S100 Calcium binding protein A10 (S100A10)	Sense	cacaccaaaatgccatctca	389	2.5	2.1
	Antisense	cttctatgggggaagctgtg			
Nidogen 2 (NID2)	Sense	gcttacgaggaggtcaaacg	500	6.8	2.9
	Antisense	ttcacccggaaggtattcag			
Transmembrane 4 superfamily member 1 (TM4SF1)	Sense	tcgcggctaatattttgctt	500	3.2	1.9
	Antisense	gcctccaagcactccattta			
Chondoitin sulfate proteoglycan 2 (CSPG2)	Sense	gaaccaaattatggggcaga	400	3.3	3.0
	Antisense	ctcccaatccttcgtcgata			
Insulin-like growth factor binding protein 3 precursor (IGFBP3)	Sense	gggtaggcacgttgtaggaa	603	-2.8	-2.8
	Antisese	gtgaggctggctaagaatgc			
Metallothionine (hMT-Ie)	Sense	cagagggtctctgggtttca	400	4.2	3.3
	Antisense	gccccatgtcctctcactaa			

* Average intensity of Adhesion/Normal peritoneal fibroblast gene expression data from patient 1(P1) and 2 (P2) presented in Table 2. Minus (-) sign indicate fold decrease in intensity in the adhesion fibroblasts. ** Ratios of Adhesion/Normal mean values from 4 patients.

**Table 4:** Expression profiles of genes in the adhesion vs. normal peritoneal fibroblasts and the effects of TGF-β1 or hypoxia on the expression level of genes in the normal peritoneal fibroblasts

**Transcripts**	**Adhesion/Normal fibroblasts **(Gene Filter & RT-PCR)	**TGF-β1 Effects **(RT-PCR)	**Hypoxia Effects **(RT-PCR)
COL4A2	↑	↑	↑
S100A10	↑	↑	↑
NID2	↑	**—**	**ND**
TM4SF1	↑	**—**	**ND**
CSPG2	↑	↑	**—**
IGFBP3	↓	**—**	**ND**
hMT-Ie	↑	↑	↑

↑ = Up regulation (p < 0.05); ↓ = Down Regulation (p < 0.05); **— **= No Change **ND **= Not determined

Initially cDNA of interests were amplified from normal peritoneal fibroblasts at different (25 to 35) PCR cycles. PCR products were subjected to agarose gel electrophoresis. Molecular weight marker (100 bp DNA ladder; Life Technology) were also loaded in adjacent lanes. DNA in the gel were stained with 1:10,000 dilution of SYBR Green I dye (Molecular Probes, Inc., Eugene, OR) and photographed using a DC 120 Kodak digital camera (Eastman Kodak, Rochester, NY) for the verification of size and analysis of band intensity using Image J software . Band intensities were plotted to determine the linearity of PCR reactions for the amplification of target transcripts. Target cDNA were amplified by PCR from normal and peritoneal fibroblasts at specific PCR cycle within its linear range of amplification. Total RNA samples from normal and adhesion fibroblasts of 4 patients (included RNA from normal and adhesion fibroblasts of two patients used for the gene filter experiments) were used for the RT-PCR experiments. Optical densities obtained from amplicons of 4 patients (1 normal and 1 adhesion fibroblast RNA sample per patient) were used to derive mean ± standard error of mean values representing relative levels of each mRNA species in normal and adhesion fibroblasts.

### Effects of TGF-β_1 _or hypoxia on gene expression pattern

Effects of TGF-β1 or hypoxic conditions on the steady state levels of specific gene transcripts in the normal peritoneal fibroblasts were also studied to examine the possibility of adhesion causing factors potentiating the gene expression pattern in the normal fibroblasts similar to adhesion fibroblasts. Normal peritoneal fibroblasts were cultured in six well culture plates (FALCON). When confluent, monolayer of cells in culture were exposed to 1 ng/ml TGF-β_1 _(Sigma Chemical Company, St. Louis, MO) or hypoxia (2% Oxygen) for 24 h. Control plates were cultured for the same duration in absence of TGF-β1 or hypoxia. Total RNA was isolated from the control, TGF-β1 and hypoxia treated cells and subjected to RT-PCR reactions as described above to determine relative levels of 18S ribosomal subunit and gene specific transcripts in the control and treated cells. RT-PCR experiments were conducted twice with the normal peritoneal fibroblasts isolated from 3 patients to have six control, six TGF-β1 treated and six hypoxia exposed amplicons. This included normal fibroblasts from one new patient and two patients that were used exclusively for RT-PCR experiments for the confirmation of gene array data. Optical densities of amplicons from six control or treated cells per mRNA species were used to derive the mean ± standard error of mean values for comparison.

### Statistical analysis

Band intensity value of each RT-PCR experiment (normal, adhesion or treated fibroblasts) was used to derive Mean ± Standard error of Mean using Statview 4.5 software (Abacus Concepts, Berkley, CA). Differences between Means were tested for significance by one-way analysis of variance with the specific post hoc test using the same software to compare differences in the steady state levels of different mRNA species.

## Results

### Expression pattern of genes between adhesion and normal peritoneal fibroblasts

**Figure 1:**
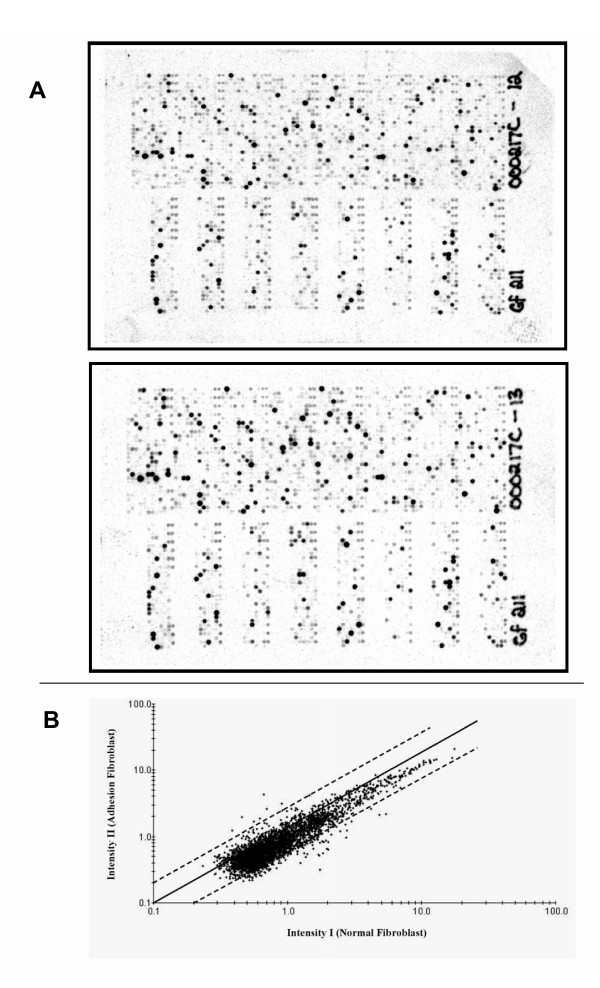
**Images depicting radioactive signals from GF211 filters hybridized with radiolabeled cDNA. **Gene filters were hybridized with ^33^P labeled cDNA from normal peritoneal fibroblasts or fibroblasts from adhesion tissue. Unbound signals were washed and relative radioactive signal intensities were detected using a Phosphoroimager as described in the Methods. **A. **Tiff images of radioactive signals from individual gene spots of filters hybridized with normal (above) and adhesion fibroblasts, both isolated from Patient 1. **B. **Scatter plot showing signal intensities from normal peritoneal (Intensity I) and adhesion (Intensity II) fibroblasts. Dotted lines indicate a two fold changes in hybridization intensities from the median (solid line).

Hybridization intensities of radio labeled cDNA from normal or adhesion fibroblasts from both the patients were different when analyzed using Pathway software. Comparison of hybridization intensities from individual gene spots between normal and adhesion fibroblast RNA (Figure 1) demonstrated that the expression levels of ~4% genes were >1.5 fold different. BLAST search of the accession number of genes from the list provided by the manufacturer showed that genes with altered expression level between normal and adhesion fibroblasts are reported to be involved in cell adhesion and migration; transformation, transcription, translation and growth factors as well as cytokines and signaling molecules.

Gene filter data from two patients showed similar expression pattern of collagen type 1 (alpha 2), Collagen type III (alpha 1), fibronectin 1, Matrix metalloproteinase-1 (MMP-1), Transforming Growth Factor beta-1 (TGF-β1), TGF-β2 and tissue plasminogen activator as reported earlier using multiplex PCR technique (Table-1). Signal intensities representing TGF-β3 (gi:22531293), TGF-β III Receptor (gi:26251745), VEGF-A (gi:2565322), VEGF-B (gi:39725673) and VEGF-C (gi:19924300) expression levels were respectively 1.6, 1.5, 1.9, 1.3 and 1.3 fold (average values from two patients) lower in the adhesion compared to normal fibroblasts. No spots for antiapoptotic bcl-2 and proapoptotic bax were present in GF211 filters. Signal intensities representing anti apoptotic molecule bcl-2 related gene bfl-1 (gi: 14574570) and pro-apoptotic molecule p53 (gi:796812) were higher (Table 2) in adhesion compared to normal fibroblasts. Expression levels of proapoptotic molecule bad (gi: 14670387) and bak1 (gi: 33457353) were not different between normal and adhesion fibroblasts. A list of additional genes that are differentially expressed between normal and adhesion fibroblasts and known to be involved in apoptosis as well as cell adhesion, proliferation and migration are listed in Table 2.

**Table 1:** Ratios of signal intensities from adhesion and normal peritoneal fibroblasts detected from gene filters representing relative expression level of genes in patient 1 (P1) and 2 (P2).

**Gene**	**Accession Number**	**P1 (A/N)**	**P2 (A/N)**	Reference*
Collagen Type I (alpha 2)	gi:48762933	1.4	1.5	[4,15,53]
Collagen Type III (alpha 1)	gi:15149480	2.0	1.7	[15]
Fibronectin 1	gi:53791222	1.5	1.2	[4,15]
MMP-1	gi:13027798	1.6	1.4	[4]
TGF-β1	gi:10863872	1.4	1.7	[4,15]
TGF-β2	gi:339549	1.5	1.3	[4]
tPA	gi:2161467	-1.5	-2.0	[8]

Minus (-) sign represents lower signal intensity in adhesion (A) compared to normal (N) fibroblasts (gene filter data)

* Citations reporting expression levels of respective genes in fibroblasts from normal human peritoneum and adhesion using multiplex PCR technique

**Figure 2:**
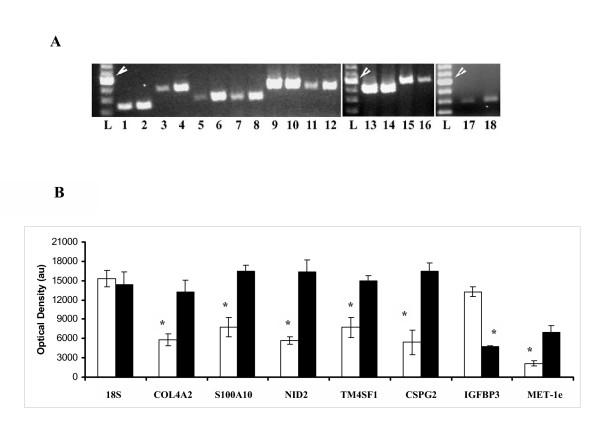
**Relative abundance of specific mRNA species in the normal and adhesion fibroblasts. **Genes differentially expressed between the normal and adhesion fibroblasts, as identified by gene filter experiments, were amplified by the RT-PCR technique at 26 PCR cycle. PCR products (20 μl) were subjected to electrophoresis, stained with fluorescent dye, photographed and optical density determined as described in Methods. **A**: Representative gel showing amplicons from normal (odd lane numbers) and adhesion (even lane numbers) fibroblasts. Lanes **1 **&**2, 3 **&**4; 5 **&**6; 7 **&**8; 9 **&**10 **and **11 **&**12 **show RT-PCR products from COL4A2; NID2; CSPG2; S100A10; 18S ribosomal subunit and TM4SF1 mRNA respectively. Lanes **13 **&**14; 15 **&**16 **and **17 **&**18 **show RT-PCR products from 18S ribosomal subunit, IGFBP3 precursor and MET-1e mRNA respectively. **L: **Lanes loaded each with 7 μl of 100 bp DNA ladder. The 600 bp band of the ladder is shown by arrow head. **B**. Histogram showing mean and standard error of mean values of optical densities derived from amplicons of specific genes (x axis) from normal (empty bars) and adhesion (filled bars) fibroblasts isolated from 4 patients as described in Methods. *Significantly different (*p *< 0.05) between normal and adhesion fibroblasts.

Semiquantitative RT-PCR experiments (Figure 2) conducted to verify expression pattern of specific genes from the gene filter experiments that were not studied earlier in the peritoneal fibroblasts confirmed higher expression (p < 0.05) of Collagen type IV (alpha 2) chain (COL4A2), S100 Calcium binding protein A10 (S100A10), Nidogen 2 (NID2), Transmembrane 4 superfamily member 1 (TM4SF1), Chondroitin sulfate proteoglycan 2 (CSPG2) and Metallothioneine (hMT-Ie) in adhesion compared to normal fibroblasts. The semiquantitative RT-PCR experiments also confirmed lower expression levels of Insulin-like growth factor binding protein 3 precursor (IGFBP3) mRNA in the adhesion compared to normal peritoneal fibroblasts. Transcript levels of 18S ribosomal subunit estimated by RT-PCR method was not significantly different between fibroblasts isolated from normal and adhesion peritoneum (Figure 2 and Table 3).

### Effects of TGF-β1 or hypoxia on the expression levels of specific genes in the normal peritoneal fibroblasts

**Figure 3:**
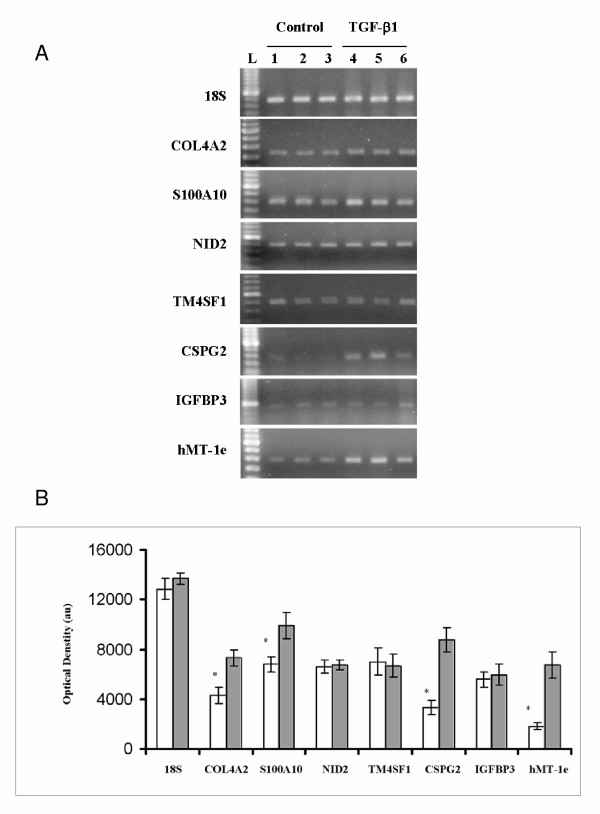
**Effects of TGF-β1 on the steady state levels of specific mRNA species in normal peritoneal fibroblasts. **Normal peritoneal fibroblasts were cultured for 24 h in absence or presence of TGF-β1 and total RNA from cells were examined for the steady-state levels of different mRNA species by semiquantitative RT-PCR technique as described in Methods. **A**. Representative gels showing amplicons generated by RT-PCR from specific gene transcripts (denoted on the left of the panel) from control (lanes 1, 2 and 3) and TGF-β1 (lanes 4, 5 and 6) treated cells. Complementary DNA for all genes except IGFBP3 precursor was amplified at 26 PCR cycles. IGFBP3 precursor transcripts were amplified at 25 cycles. **L **Lane loaded with 100 bp DNA ladder. **B **Histogram showing mean and standard errors of mean of optical densities from amplicons representing specific mRNA species (x axis). The RT-PCR experiments were conducted twice from normal and peritoneal isolated from 3 patients to obtain OD values of six amplicons from control (empty bars) or treated (shaded bars) fibroblasts statistical analysis. * Significantly different from control conditions at *p *< 0.05.

**Figure 4:**
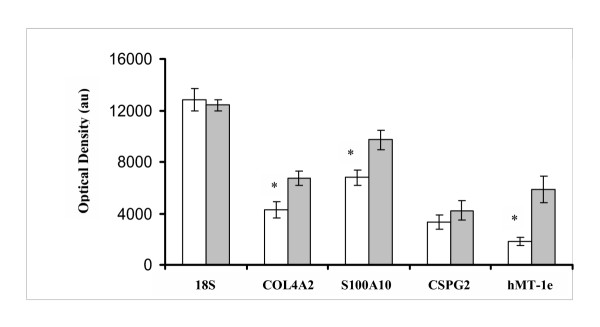
**Effects of hypoxia on the steady state levels of specific genes in normal peritoneal fibroblasts. **Normal peritoneal fibroblasts were cultured for 24 h in normoxic and hypoxic conditions and total RNA from cells were examined to determine the steady state levels of specific transcripts as described in Methods. Complementary DNA for all genes was amplified at 26 PCR cycles. Histogram showing mean and standard errors of mean of optical densities of amplicons representing specific mRNA species (x axis) from control (empty bars) or hypoxia exposed cells (shaded bars) from 3 patients. The RT-PCR experiments were conducted twice to obtain OD values of six amplicons from normoxic or hypoxic fibroblasts for statistical analysis. Images of gels with amplicons from cells treated with hypoxia are not shown. * Significantly different from control conditions at *p *< 0.05.

Exposure to TGF-β_1 _or hypoxic conditions for 24 h altered expression levels of specific genes in the normal peritoneal fibroblasts as evidenced by semiquantitative RT-PCR. Transcript levels of COL4A2, S100A10, CSPG2 and hMT-Ie were up regulated by TGF-β1 in the normal peritoneal fibroblasts (Figure 3), whereas transcript levels of NID2, TM4SF1, and IGFBP3 were not altered by TGF-β1 treatment. Hypoxic conditions elevated expression levels of COL4A2, S100A10 and hMT-Ie transcripts in the normal peritoneal fibroblasts (Figure 4). Transcript levels of CSPG2 were not significantly altered by hypoxia.

## Discussion

We present evidence that the expression pattern of large number of genes differ between the fibroblasts isolated from adhesion tissues and normal human peritoneal supporting the notion that adhesion fibroblasts may attain a different phenotype following peritoneal injury. Genes that displayed altered expression levels in this transition included those involved in cell proliferation, differentiation, signaling molecules, transcription and translation factors, proteolysis and cytokines. Results indicate that fibroblasts from adhesion tissue may perceive and respond to external and internal cues differently than those residing in normal human peritoneum. We attempted to decipher the functional consequences of altered gene expression pattern in the adhesion fibroblast to further elucidate the mechanism of adhesion formation and point to additional ways adhesion development may be restrained.

Expression pattern of genes in the fibroblasts from normal and pathological sites are shown to be different also in earlier studies [17]. More relevant to the present study are the reports [4,8] on the mRNA levels of human type I collagen (alpha 2), fibronectin 1, MMP-1, TIMP-1, TGF-β1, TGF-β2, IL-10, PAI-1, tPA and COX-2 in adhesion and normal peritoneal fibroblasts from humans estimated by multiplex PCR technique. Gene filter data from two patients also showed similar pattern of collagen, type 1 (alpha 2), fibronectin 1, MMP-1, TGF-β1, TGF-β2 and tPA mRNA levels in the normal and adhesion fibroblasts (Table 1). Expression pattern of TIMP-1, IL-10, PAI-1, COX-2 in the adhesion and normal peritoneal fibroblasts as reported earlier [4,8,9] could not be verified by gene filter experiments because GF211 filters do not have spots representing these genes. Even so, similarities in the expression pattern of many genes between two patients (Tables 1–3) and those reported earlier using multiplex PCR technique [4,8] validate our findings. The semiquantitative RT-PCR experiments conducted to verify expression pattern of specific genes recorded from gene filter experiments show that mRNA levels of COL4A2, S100A10, nidogen-2, TM4SF1, CSPG2, MT-1e and IGFBP3 precursor indeed differ between normal and adhesion fibroblasts.

Even though expression levels of these transcripts were significantly different between normal and adhesion fibroblasts, only minor variations in the optical densities of amplicons were recorded within normal or adhesion tissues of patients of different age groups. This indicate that age dependent differences in the expression levels of genes in the fibroblasts from normal or adhesion tissues may tend to attain a relatively similar expression levels when in culture. Despite the fact that our study focused on the steady state levels of mRNA species and not on translation or posttranslational events, analysis of the functional consequences of altered expression of encoded proteins from the literature as referred below indicated that changes in the pool of these mRNA species may lead to the transformation of normal peritoneal fibroblasts into a specialized phenotype during the healing process.

COL4A2 is a major structure-defining component in all basement membranes [18] and forms a framework for the ordered aggregation of additional molecules like laminin, heparin sulphate proteoglycans, and nidogen [19]. Relatively higher levels of COL4A2 observed in the adhesion fibroblasts may enhance synthesis of basement membrane in the tissues of adhesions. As COL4A2 gene is up regulated during malignant transformation and tumor vessel proliferation [20], it is anticipated that up regulated levels of COL4A2 in the adhesion fibroblasts may aid to the formation of adhesion tissue by increasing proliferation of adhesion fibroblasts and supporting new vessel formation for the nourishments of growing tissue.

S100A10 proteins interact with Annexin A2 forming a heterotetrameric structure AIIt; that dock into the cell membrane promoting F-actin reorganization and cell migration [21]. AIIt also colocalizes with uPAR and plasminogen in the cells [22]. Heightened levels of S100A10 may enhance migration of adhesion fibroblasts by changing F-actin dynamics and influencing Cathepsin B and plasminogen machinery [23]. S100A10 also interacts with cytosolic phospholipase A2, inhibits its activity and decreases synthesis of archidonic acid [24]. Therefore, increase in S100A10 levels in the adhesion fibroblasts may deplete intracellular levels of archidonic acid and Prostaglandin E2 (PGE2) that are known to inhibit cell proliferation, collagen I synthesis, contraction of ECM and fibroblast migration [25].

Nidogen-2 (entactin-2) interacts with laminin1 P1, collagen I, collagen IV, perlecan and fibulin-2 in the extracellular space and stabilizes the basement membrane. It also interacts with α6β1 and α3β1 integrin receptors on cells [26]. Relatively higher levels of nidogen-2 secreted by adhesion fibroblasts in the extracellular space may strengthen the basement membrane and enhance integrin mediated adhesion and migration of fibroblasts into the growing tissue of adhesion.

TM4SF molecules (tetraspanins) play important roles in cell migration and in the generation of complexes with integrins functionally relevant for cell motility, tumor progression and wound healing [27]. It is proposed that tetraspanins can influence cell migration by (i) modulating integrin signaling and integrin-mediated reorganization of the cortical actin cytoskeleton; (ii) regulating compartmentalization of integrins on the cell surface or (iii) directing intracellular trafficking and recycling of integrins [27]. Therefore, heightened intercalation of TM4SF1 in the cell surface of adhesion fibroblasts may facilitate their integrin-mediated migration into the developing tissues of adhesion.

Versicans (CSPG2) are also known to influence α4β1 and α2β1 integrin mediated invasion of melanoma cells [28]. Higher CSPG2 in the fibroblasts of adhesion tissues may assist in the integrin-CSPG2 mediated migration of peritoneal fibroblasts to the site of injury and increase the number of fibroblasts by enhancing proliferation and decreasing apoptosis as evidenced in other cell types [28,29]. Veriscan interacts with hyaluronan and CD44 and increase the viscoelastic nature of the pericellular matrix, creating a highly malleable extracellular environment that supports a cell-shape change necessary for cell proliferation and migration [30].

Because MT-1e transcripts are detected in cell types that have undergone myoepithelial differentiation [31], significant differences in the MT-1e mRNA levels between adhesion and normal peritoneal fibroblasts indicate that fibroblasts in the adhesions are at different state of differentiation compared to normal peritoneum. Molecules including IL-1; IL-6, TNF-α, EGF, bFGF, glucocorticoids, LPS, and estrogen that promote post surgical adhesion formation [32-34] directly or indirectly increase MT-1 transcripts and proteins in several tissues and cell types [35]. Therefore, it is likely that these molecules may increase adhesion formation by augmenting MT-IE levels which in turn may increase proliferation, reduce cell death and confer invasiveness of adhesion fibroblasts [36].

Contrary to increase in the above mentioned mRNA species in the adhesion fibroblasts, steady state levels of IGFBP3 precursor transcript were found to be lower. Because IGFBP-3 is known to inhibit cell growth by sequestering IGF, its decreased level may enhance proliferation of adhesion fibroblasts [37]. Reduced levels of IGFBP3 mRNA are reported in the tumorigenic cells [38]. Therefore, reported lower incidence of pelvic adhesion formation in the primates on anti-estrogenic therapy [32] could be due to the antiproliferative effects of anti-estrogens mediated in part, by IGFBP-3 [39]. IGFBP-3 also induces growth inhibition and apoptosis [40]. Decrease in the levels of IGFBP-3 in the adhesion fibroblasts may promote adhesion development both by increasing proliferation and reducing apoptosis at the site of injury.

Our attempts to examine the regulatory roles of TGF-β1 and hypoxia, factors known to promote adhesion development [3], on the expression pattern of specific genes show that not all changes in the gene expression pattern between the normal and adhesion fibroblast are the function of these factors (Figure 3 and 4; Table 4). Our data show that while mRNA levels of COL4A2, S100A10 and MT-1e are elevated by both TGF-β1 and hypoxia in the human peritoneal fibroblasts, the mRNA levels of only CSPG2 is influenced by TGF-β1. Moreover, transcript levels of nidogen-2, TM4SF1 and IGFBP3 mRNA were not influenced by TGF-β1. Based on these results we hypothesize that genes that are not influenced by TGF-β1 and hypoxia in the peritoneal fibroblasts may be influenced by factors such as interleukins and TNF-α that are also known to play role in adhesion formation. Alternately, TGF-β1 and/or hypoxia may influence actions of these genes at the post transcriptional level without altering transcript levels. TGF-β1 induced up regulation of integrin α5, αv and α6 subunits in the normal human peritoneal fibroblasts without altering mRNA levels [10] is consistent with this possibility. It is also possible that TGF-β1 and hypoxia may alter expression of these genes in mesothelial and other cell types following peritoneal injury. Likewise lower levels of VEGF transcripts in adhesion fibroblasts may be compensated by its higher levels in other cell type required for angiogenesis during adhesion formation [3]. Detected lower intensity of VEGF-A isoform in the adhesion fibroblasts may also be due to the fact that spots representing this isoform do not distinguish different VEGF-A splice variants that are known to be up or down regulated during adhesion formation [16].

It is known that a new phenotype of fibroblasts is induced during wound healing. These fibroblasts, termed- myofibroblasts, express higher levels of α-smooth muscle actin and vinculin-containing fibronexus adhesion complexes [41]. Fibroblasts isolated from adhesion tissues express higher levels of α-smooth muscle actin transcripts compared to normal peritoneal fibroblast (Table-2) [42] and TGF-β1 induces formation of adhesion complex in these cells [10]. These observations in addition to the known roles of TGF-β in the development of post surgical adhesions [43] and transformation of fibroblasts into smooth muscle α-actin expressing myofibroblasts [44] imply that this cytokine may influence transformation of normal fibroblasts into a phenotype similar to myofibroblasts in the developing tissues of adhesion. Therefore, hindering this transformation may reduce adhesion formation. For instance, augmenting E prostanoid 2 (EP2) receptor pathways may be a way to reduce the incidence of adhesion formation because prostaglandin E_2 _(PGE_2_) is shown to inhibit TGF-β1 induced expression of α-SMA, production of Collagen I and the transformation of fibroblasts to myofibroblasts via EP2 signaling [45]. Additionally, adhesion formation may be reduced by *P311 *(*PTZ17*) and Interferon γ treatments, which inhibits TGF-β1 induced myofibroblast transformation [46,47].

During the course of normal wound healing, myofibroblasts disappear, possibly by apoptosis [48]. In contrast, when there is abnormal wound healing, myofibroblasts persist [49]. Data obtained in our study also indicate that adhesion fibroblasts may resist apoptosis due to anti apoptotic effects mediated by increased hMet1-e and CSPG2 levels and down regulation of IGFBP3. They may also attain a high proliferating nature due to up regulation of S100A10 and CSPG2 genes, and down regulation of IGFBP3 (Table 2). Higher proliferating and reduced apoptotic nature of adhesion fibroblasts derived from altered ratio of bcl-2 and bax expression is suggested in an earlier study [5]. It is apparent now that higher proliferative and reduced apoptotic nature of adhesion fibroblasts in human as reported earlier [5] could also derive from altered expression of hMet1-e, CSPG2, S100A10, CSPG2, IGFBP3, and the Bfl-1 that inhibits p53-induced apoptosis and is induced by cytokines TNF-α and IL-1β [50]. This altered phenotype of adhesion fibroblasts, acquired during the healing process, may lead to the accumulation of extra number of cells at the site of peritoneal injury resulting fibrosis and scar formation. Of note, one of the pivotal differences between wounds that proceed to normal scar compared with those that develop hypertrophic scars or fibrosis may be a lack of or reduced cell death [51]. Therefore, excess fibroblasts at the site of peritoneal wound healing may divert the normal process of healing towards fibrosis and adhesion. The elevated levels of p53 in the adhesion fibroblasts during this disarray, as evident from the gene filter data (Table 2), may guard against its transition towards malignancy [52].

## Conclusions

It is evident from our study that steady state levels of several genes are different between adhesion and normal peritoneal fibroblasts in human and that adhesion development may be a function of several genes. Changes in the functional interdependence of these genes at the site of injury may transform normal peritoneal fibroblast into cell type/s with altered phenotype. These cells- designated as adhesion fibroblasts may mimic previously known myofibroblasts and are highly proliferative. These cells resist apoptosis and secrete ECM molecules to renovate basement membrane. With changed expression pattern of cell surface molecules these cells may respond to intracellular signaling for migration over the fibrin clot. This altered nature of adhesion fibroblasts therefore may play a major role in the formation of the fibrous mass of adhesion-tissue that bridges adjacent and injured peritoneum. Blocking changes in the expression or function of genes necessary for this transformation of normal peritoneal fibroblasts may curtail adhesion formation. This could be achieved by the application of PGE_2_, EP_2 _blockers, interferon γ, *P311 *and applying measures to induce apoptosis in the peritoneal fibroblast at the site of injury. The obvious question – "how to maintain apoptosis at a desired level for normal peritoneal healing?" however, remains to be answered.

## List of Abbreviations

Collagen type IV (alpha 2) chain (COL4A2), Nidogen 2 (NID2), Chondroitin sulfate proteoglycan 2 (CSPG2), S100 Calcium binding protein A10 (S100A10), Transmembrane 4 superfamily member 1 (TM4SF1), Metallothioneine (hMT-Ie), Insulin-like growth factor binding protein 3 precursor (IGFBP3), Transforming growth factor (TGF), Prostaglandin E2 (PGE2), Urokinase Plasminogen activator receptor (uPAR), Annexin 2 and S100A10 complex (AIIt), tissue Plasminogen Activator (tPA), Plasminogen Activator Inhibitor (PAI), Cyclooxigenase (COX), Matrix metalloproteinase (MMP), Tissue Inhibitor of Metalloproteinase (TIMP), Interferon γ (IFN-γ), IL (Interleukin).

## Authors' Contributions

GMS and MPD were responsible for the isolation of peritoneal fibroblasts from normal peritoneum and adhesion tissues as well as establishing hypoxia chambers. MPD provided patient information and valuable suggestions during writing the manuscript. UKR performed microarray and semiquantitative RT-PCR experiments, analyzed the data and wrote the manuscript.

## References

[B1] Diamond MP (1996). Reduction of adhesions after uterine myomectomy by Seprafilm membrane (HAL-F): a blinded, prospective, randomized, multicenter clinical study. Seprafilm Adhesion Study Group. Fertil Steril.

[B2] Ellis H, Moran BJ, Thompson JN, Parker MC, Wilson MS, Menzies D, McGuire A, Lower AM, Hawthorn RJ, O'Brien F (1999). Adhesion-related hospital readmissions after abdominal and pelvic surgery: a retrospective cohort study. Lancet.

[B3] Chegini N (2002). Peritoneal molecular environment, adhesion formation and clinical implication. Front Biosci.

[B4] Saed GM, Zhang W, Diamond MP (2001). Molecular characterization of fibroblasts isolated from human peritoneum and adhesions. Fertil Steril.

[B5] Saed GM, Diamond MP (2002). Apoptosis and proliferation of human peritoneal fibroblasts in response to hypoxia. Fertil Steril.

[B6] Saed GM, Diamond MP (2002). Hypoxia-induced irreversible up-regulation of type I collagen and transforming growth factor-beta1 in human peritoneal fibroblasts. Fertil Steril.

[B7] Saed GM, Diamond MP (2003). Effect of glucose on the expression of type I collagen and transforming growth factor-beta1 in cultured human peritoneal fibroblasts. Fertil Steril.

[B8] Saed GM, Diamond MP (2003). Modulation of the expression of tissue plasminogen activator and its inhibitor by hypoxia in human peritoneal and adhesion fibroblasts. Fertil Steril.

[B9] Saed GM, Munkarah AR, Diamond MP (2003). Cyclooxygenase-2 is expressed in human fibroblasts isolated from intraperitoneal adhesions but not from normal peritoneal tissues. Fertil Steril.

[B10] Rout UK, Saed GM, Diamond MP (2002). Transforming growth factor-beta1 modulates expression of adhesion and cytoskeletal proteins in human peritoneal fibroblasts. Fertil Steril.

[B11] Falanga V (1998). Wound healing and chronic wounds. J Cutan Med Surg.

[B12] Gailit J, Clarke C, Newman D, Tonnesen MG, Mosesson MW, Clark RA (1997). Human fibroblasts bind directly to fibrinogen at RGD sites through integrin alpha(v)beta3. Exp Cell Res.

[B13] Saed GM, Zhang W, Chegini N, Holmdahl L, Diamond MP (2000). Transforming growth factor beta isoforms production by human peritoneal mesothelial cells after exposure to hypoxia. Am J Reprod Immunol.

[B14] Gago LA, Saed GM, Chauhan S, Elhammady EF, Diamond MP (2003). Seprafilm (modified hyaluronic acid and carboxymethylcellulose) acts as a physical barrier. Fertil Steril.

[B15] Saed GM, Kruger M, Diamond MP (2004). Expression of transforming growth factor-beta and extracellular matrix by human peritoneal mesothelial cells and by fibroblasts from normal peritoneum and adhesions: effect of Tisseel. Wound Repair Regen.

[B16] Rout UK, Oommen K, Diamond MP (2000). Altered expressions of VEGF mRNA splice variants during progression of uterine-peritoneal adhesions in the rat. Am J Reprod Immunol.

[B17] Fries KM, Blieden T, Looney RJ, Sempowski GD, Silvera MR, Willis RA, Phipps RP (1994). Evidence of fibroblast heterogeneity and the role of fibroblast subpopulations in fibrosis. Clin Immunol Immunopathol.

[B18] Timpl R (1989). Structure and biological activity of basement membrane proteins. Eur J Biochem.

[B19] Yurchenco PD, Schittny JC (1990). Molecular architecture of basement membranes. Faseb J.

[B20] van den Boom J, Wolter M, Kuick R, Misek DE, Youkilis AS, Wechsler DS, Sommer C, Reifenberger G, Hanash SM (2003). Characterization of gene expression profiles associated with glioma progression using oligonucleotide-based microarray analysis and real-time reverse transcription-polymerase chain reaction. Am J Pathol.

[B21] Zokas L, Glenney JR (1987). The calpactin light chain is tightly linked to the cytoskeletal form of calpactin I: studies using monoclonal antibodies to calpactin subunits. J Cell Biol.

[B22] Zhang L, Fogg DK, Waisman DM (2004). RNA interference-mediated silencing of the S100A10 gene attenuates plasmin generation and invasiveness of Colo 222 colorectal cancer cells. J Biol Chem.

[B23] Choi KS, Fogg DK, Yoon CS, Waisman DM (2003). p11 regulates extracellular plasmin production and invasiveness of HT1080 fibrosarcoma cells. Faseb J.

[B24] Wu T, Angus CW, Yao XL, Logun C, Shelhamer JH (1997). P11, a unique member of the S100 family of calcium-binding proteins, interacts with and inhibits the activity of the 85-kDa cytosolic phospholipase A2. J Biol Chem.

[B25] Kohyama T, Ertl RF, Valenti V, Spurzem J, Kawamoto M, Nakamura Y, Veys T, Allegra L, Romberger D, Rennard SI (2001). Prostaglandin E(2) inhibits fibroblast chemotaxis. Am J Physiol Lung Cell Mol Physiol.

[B26] Kohfeldt E, Sasaki T, Gohring W, Timpl R (1998). Nidogen-2: a new basement membrane protein with diverse binding properties. J Mol Biol.

[B27] Berditchevski F (2001). Complexes of tetraspanins with integrins: more than meets the eye. J Cell Sci.

[B28] Iida J, Meijne AM, Knutson JR, Furcht LT, McCarthy JB (1996). Cell surface chondroitin sulfate proteoglycans in tumor cell adhesion, motility and invasion. Semin Cancer Biol.

[B29] Wight TN (2002). Versican: a versatile extracellular matrix proteoglycan in cell biology. Curr Opin Cell Biol.

[B30] Lee GM, Johnstone B, Jacobson K, Caterson B (1993). The dynamic structure of the pericellular matrix on living cells. J Cell Biol.

[B31] Dempsey PJ, de Kretser TA, Brown RW, Whitehead RH, Jose DG (1986). A monoclonal antibody CIBr17 recognizes a myoepithelium-specific antigen in human mammary gland. Int J Cancer.

[B32] Grow DR, Coddington CC, Hsiu JG, Mikich Y, Hodgen GD (1996). Role of hypoestrogenism or sex steroid antagonism in adhesion formation after myometrial surgery in primates. Fertil Steril.

[B33] Wiczyk HP, Grow DR, Adams LA, O'Shea DL, Reece MT (1998). Pelvic adhesions contain sex steroid receptors and produce angiogenesis growth factors. Fertil Steril.

[B34] Cheong YC, Shelton JB, Laird SM, Richmond M, Kudesia G, Li TC, Ledger WL (2002). IL-1, IL-6 and TNF-alpha concentrations in the peritoneal fluid of women with pelvic adhesions. Hum Reprod.

[B35] Carrasco J, Hernandez J, Bluethmann H, Hidalgo J (1998). Interleukin-6 and tumor necrosis factor-alpha type 1 receptor deficient mice reveal a role of IL-6 and TNF-alpha on brain metallothionein-I and -III regulation. Brain Res Mol Brain Res.

[B36] Theocharis SE, Margeli AP, Koutselinis A (2003). Metallothionein: a multifunctional protein from toxicity to cancer. Int J Biol Markers.

[B37] Valentinis B, Bhala A, DeAngelis T, Baserga R, Cohen P (1995). The human insulin-like growth factor (IGF) binding protein-3 inhibits the growth of fibroblasts with a targeted disruption of the IGF-I receptor gene. Mol Endocrinol.

[B38] Nishizuka S, Winokur ST, Simon M, Martin J, Tsujimoto H, Stanbridge EJ (2001). Oligonucleotide microarray expression analysis of genes whose expression is correlated with tumorigenic and non-tumorigenic phenotype of HeLa x human fibroblast hybrid cells. Cancer Lett.

[B39] Huynh H, Yang X, Pollak M (1996). Estradiol and antiestrogens regulate a growth inhibitory insulin-like growth factor binding protein 3 autocrine loop in human breast cancer cells. J Biol Chem.

[B40] Butt AJ, Fraley KA, Firth SM, Baxter RC (2002). IGF-binding protein-3-induced growth inhibition and apoptosis do not require cell surface binding and nuclear translocation in human breast cancer cells. Endocrinology.

[B41] Roy SG, Nozaki Y, Phan SH (2001). Regulation of alpha-smooth muscle actin gene expression in myofibroblast differentiation from rat lung fibroblasts. Int J Biochem Cell Biol.

[B42] Saed GM, Diamond MP (2004). Differential expression of alpha smooth muscle cell actin in human fibroblasts isolated from intraperitoneal adhesions and normal peritoneal tissues. Fertil Steril.

[B43] Falanga V, Zhou L, Yufit T (2002). Low oxygen tension stimulates collagen synthesis and COL1A1 transcription through the action of TGF-beta1. J Cell Physiol.

[B44] Vaughan MB, Howard EW, Tomasek JJ (2000). Transforming growth factor-beta1 promotes the morphological and functional differentiation of the myofibroblast. Exp Cell Res.

[B45] Kolodsick JE, Peters-Golden M, Larios J, Toews GB, Thannickal VJ, Moore BB (2003). Prostaglandin E2 inhibits fibroblast to myofibroblast transition via E. prostanoid receptor 2 signaling and cyclic adenosine monophosphate elevation. Am J Respir Cell Mol Biol.

[B46] Hansson GK, Hellstrand M, Rymo L, Rubbia L, Gabbiani G (1989). Interferon gamma inhibits both proliferation and expression of differentiation-specific alpha-smooth muscle actin in arterial smooth muscle cells. J Exp Med.

[B47] Pan D, Zhe X, Jakkaraju S, Taylor GA, Schuger L (2002). P311 induces a TGF-beta1-independent, nonfibrogenic myofibroblast phenotype. J Clin Invest.

[B48] Zhang HY, Phan SH (1999). Inhibition of myofibroblast apoptosis by transforming growth factor beta(1). Am J Respir Cell Mol Biol.

[B49] Chipev CC, Simman R, Hatch G, Katz AE, Siegel DM, Simon M (2000). Myofibroblast phenotype and apoptosis in keloid and palmar fibroblasts in vitro. Cell Death Differ.

[B50] Yoon HS, Hong SH, Kang HJ, Ko BK, Ahn SH, Huh JR (2003). Bfl-1 gene expression in breast cancer: its relationship with other prognostic factors. J Korean Med Sci.

[B51] Desmouliere A, Badid C, Bochaton-Piallat ML, Gabbiani G (1997). Apoptosis during wound healing, fibrocontractive diseases and vascular wall injury. Int J Biochem Cell Biol.

[B52] Levine AJ (1997). p53, the cellular gatekeeper for growth and division. Cell.

[B53] Diamond MP, El-Hammady E, Wang R, Saed G (2002). Metabolic regulation of collagen I in fibroblasts isolated from normal peritoneum and adhesions by dichloroacetic acid. Am J Obstet Gynecol.

[B54] de Nadal E, Casadome L, Posas F (2003). Targeting the MEF2-like transcription factor Smp1 by the stress-activated Hog1 mitogen-activated protein kinase. Mol Cell Biol.

[B55] Kannengiesser C, Avril MF, Spatz A, Laud K, Lenoir GM, Bressac-de-Paillerets B (2003). CDKN2A as a uveal and cutaneous melanoma susceptibility gene. Genes Chromosomes Cancer.

[B56] Suzuki S, Suzuki N, Mirtsos C, Horacek T, Lye E, Noh SK, Ho A, Bouchard D, Mak TW, Yeh WC (2003). Nur77 as a survival factor in tumor necrosis factor signaling. Proc Natl Acad Sci U S A.

[B57] Yamazaki M, Matsuo R, Fukazawa Y, Ozawa F, Inokuchi K (2001). Regulated expression of an actin-associated protein, synaptopodin, during long-term potentiation. J Neurochem.

[B58] Samarin S, Romero S, Kocks C, Didry D, Pantaloni D, Carlier MF (2003). How VASP enhances actin-based motility. J Cell Biol.

[B59] Oh Y, Nagalla SR, Yamanaka Y, Kim HS, Wilson E, Rosenfeld RG (1996). Synthesis and characterization of insulin-like growth factor-binding protein (IGFBP)-7. Recombinant human mac25 protein specifically binds IGF-I and -II. J Biol Chem.

[B60] Iocono JA, Colleran KR, Remick DG, Gillespie BW, Ehrlich HP, Garner WL (2000). Interleukin-8 levels and activity in delayed-healing human thermal wounds. Wound Repair Regen.

[B61] Plzak J, Betka J, Smetana K, Chovanec M, Kaltner H, Andre S, Kodet R, Gabius HJ (2004). Galectin-3 – an emerging prognostic indicator in advanced head and neck carcinoma. Eur J Cancer.

[B62] Frossard JL, Rubbia-Brandt L, Wallig MA, Benathan M, Ott T, Morel P, Hadengue A, Suter S, Willecke K, Chanson M (2003). Severe acute pancreatitis and reduced acinar cell apoptosis in the exocrine pancreas of mice deficient for the Cx32 gene. Gastroenterology.

[B63] Nachtigal P, Gojova A, Semecky V (2001). The role of epithelial and vascular-endothelial cadherin in the differentiation and maintenance of tissue integrity. Acta Medica (Hradec Kralove).

[B64] Tsuda H, Ohshima Y, Nomoto H, Fujita K, Matsuda E, Iigo M, Takasuka N, Moore MA (2004). Cancer prevention by natural compounds. Drug Metab Pharmacokinet.

[B65] Tong Z, Wu X, Kehrer JP (2003). Increased expression of the lipocalin 24p3 as an apoptotic mechanism for MK886. Biochem J.

[B66] Horiuchi A, Nikaido T, Taniguchi S, Fujii S (1999). Possible role of calponin h1 as a tumor suppressor in human uterine leiomyosarcoma. J Natl Cancer Inst.

[B67] D'Silva NJ, Mitra RS, Zhang Z, Kurnit DM, Babcock CR, Polverini PJ, Carey TE (2003). Rap1, a small GTP-binding protein is upregulated during arrest of proliferation in human keratinocytes. J Cell Physiol.

[B68] Zhou CQ, Liu S, Xue LY, Wang YH, Zhu HX, Lu N, Xu NZ (2003). Down-regulation of gamma-synuclein in human esophageal squamous cell carcinoma. World J Gastroenterol.

